# Analysis of Necessary Support in the 2011 Great East Japan Earthquake Disaster Area

**DOI:** 10.3390/ijerph17103475

**Published:** 2020-05-16

**Authors:** Moeka Harada, Kazuko Ishikawa-Takata, Nobuyo Tsuboyama-Kasaoka

**Affiliations:** 1 Section of Global Disaster Nutrition, International Center for Nutrition and Information, National Institute of Health and Nutrition, National Institutes of Biomedical Innovation, Health and Nutrition, Tokyo 162-8636, Japan; harada-mo@tokyo-kasei.ac.jp; 2 Department of Food and Nutrition, Faculty of Home Economics, Tokyo Kasei University, Tokyo 173-8602, Japan; 3 Division of Nutritional Epidemiology and Shokuiku, National Institute of Health and Nutrition, National Institutes of Biomedical Innovation, Health and Nutrition, Tokyo 162-8636, Japan; kazu@nibiohn.go.jp

**Keywords:** disaster, nutrition, health, food, dietitian

## Abstract

Altogether, 1588 dietitians were dispatched from the Japan Dietetic Association (JDA) to a disaster area for the first time on a nationwide scale following the 2011 Great East Japan Earthquake. Various studies have been conducted based on the activity reports, but the support that the disaster area requested was not documented. The purpose of this study is to identify the support that was needed in the disaster area. Therefore, we investigated the necessary support desired by dietitians who lived in the disaster areas. Questionnaires were sent to 1911 dietitians who were members of the JDA and lived in 3 affected prefectures in August 2012. In total, 435 dietitians (22.8%) completed the questionnaire. Among the questions on the questionnaire, we analyzed answers to the open-ended question: “Please write freely about the support that you wanted at the time of the disaster” (*n* = 332). Using qualitative descriptive analysis, we extracted data from the answers and categorized and labeled them into similar groups. These groups were divided into four categories: (1) “goods,” (2) “establishing a system in advance of a large-scale disaster,” (3) “information,” and (4) “human resources.” To provide “goods,” “information,” and “human resources” to the disaster area smoothly, it is important to plan a “system” in advance of large-scale disasters.

## 1. Introduction

In recent years, natural hazards, such as earthquakes, typhoons, and hurricanes, have occurred around the world. Natural hazards cascade to cause disasters [1]. For example, earthquakes trigger a cascade of disasters such as building collapses, cracks, tsunamis, etc. 

After such a cascading disaster, the living environment of those who are affected often deteriorates. Necessities such as electricity, gas, and water cease, and roads may be damaged due to the collapse of buildings and landslides. It is not unexpected that dietary conditions may worsen. Several studies demonstrated that food provided to emergency shelters in Japan consisted mainly of carbohydrate-rich foods, such as rice balls, bread, and instant noodles [2,3,4]. It has also been reported that the worsening of dietary conditions following a disaster induces a deterioration in the health condition of survivors. 

The Great East Japan Earthquake hit the Tohoku district of Japan on 11 March 2011. It was reported that 19,689 were killed, 2563 were missing, and 6233 were injured (as of 8 March 2019) [5]. This earthquake is a typical cascading disaster. It triggered building collapses, a tsunami, and a nuclear reactor meltdown. Many survivors were forced to live in emergency shelters where the provision of food consisted mainly of carbohydrate-rich foods and the nutritional balance was poor, similar to the previous disaster [4]. Furthermore, the nutritional balance still did not improve even 1 month after the disaster, and this was expected to continue [6]. Therefore, the Ministry of Health, Labor and Welfare (MHLW) published Nutritional Reference Values for meal provision in emergency shelters [7]. In addition, the MHLW asked the Japan Dietetic Association (JDA) to assist with supplying food and nutrition in the disaster areas on 22 March 2011. The JDA dispatched registered dietitians and general dietitians (RDs) to the disaster areas for the first time on a national scale. 

Support activities by dispatched RDs has been documented [8]. The study reported that the frequency of “Meeting” for RDs was the highest to share information with other supporters. Additionally, the frequency of “dietary/nutritional assessment” was high. In the analysis of “What I Thought Today” reported by dispatched RDs, it was recorded that they felt anxiety, tension, and discomfort [9]. On the other hand, the analysis found that dispatched RDs felt needed in the disaster areas. The usefulness of RDs has been proven in emergency shelters. We reported that menu creation by RDs improved the diets in emergency shelters [10]. In addition, the benefits as seen from the viewpoint of RDs in the disaster area have been assessed. The benefits included the usefulness of the RDs’ skills during disasters and the fact that their assistance provided mental support [11]. On the other hand, it was documented that the assistance provided was not necessarily consistent with needs, a problematic point [11]. However, the support that RDs in the disaster area actually needed has not been documented. 

In the present study, we document the support that was needed for RDs in the disaster area. The findings of this study can be used to clarify the ways in which we can make the necessary preparations and plan for support activities in future cascading disasters.

## 2. Materials and Methods 

### 2.1. Setting and Participants

In August 2012, survey questionnaires were sent to 1911 RDs who belonged to the JDA and lived in the 3 most affected prefectures (Iwate, Miyagi, and Fukushima). Letters were sent to the same RDs in October 2012 to remind them to complete the survey. The objective of the study and the confidentiality of the data were described in the letters. Return of the questionnaire implied consent by the RDs (participants) to participate in the study. 

The questionnaire was self-administered and included a total of 157 items in maximum. The questionnaire was composed of the following sections: (1) questions about the characteristics of participants, (2) questions about the recognition and usage of some tools provided by the Japanese government, (3) questions about participant’s job in their facility for each period following the disaster, (4) questions about the support activities in emergency shelters for each period following the disaster, (5) questions about the support activities in survivors’ homes for each period following the disaster, and (6) open-ended questions. 

### 2.2. Data Analysis

Data were qualitatively and inductively analyzed through use of the qualitative descriptive analysis method. As an exploration of data organization, this analysis approach uses thematic analysis [12]. The qualitative descriptive analysis eliminates the subjectivity of the analyst and lets the data speak for itself. This analysis also encompasses everything from methods of data collection and organization to problem-solving, extending beyond the simple classification undertaken in thematic analysis. 

Specifically, this method involves classifying data while identifying similarities in the dataset. After completing this initial step, the condensed meaning units were then labeled with a code, and the subcategories were created. The code is an attempt to capture the primary content or essence of an extracted segment of data [13]. A main category is a recurrent thread of underlying meaning running through codes and categories.

**Figure 1:**
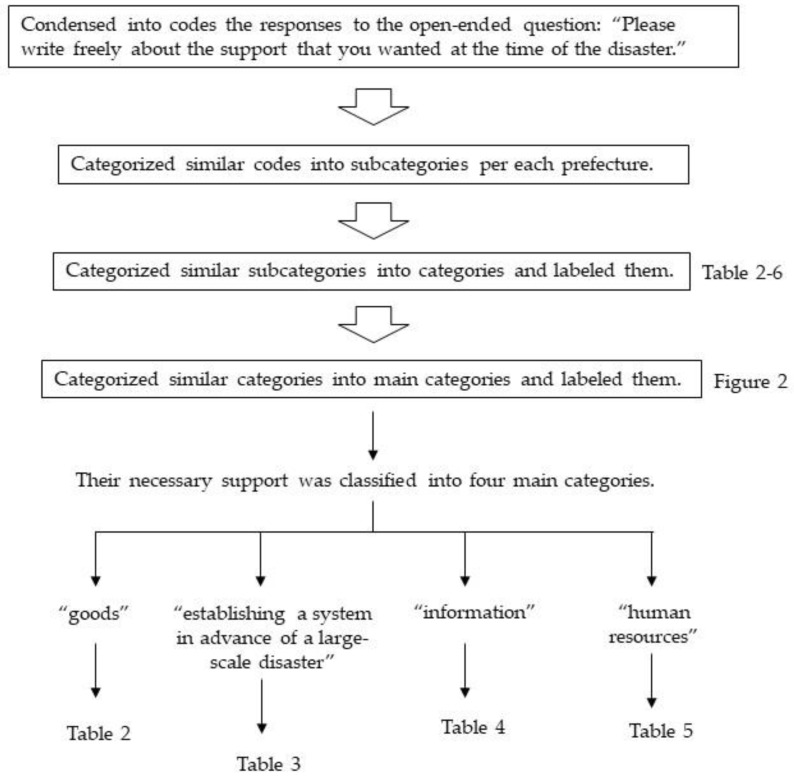
Procedure for analysis by qualitative descriptive analysis.

Participants were included in the analysis if they answered the question with the open-ended question “Please write freely about the support that you wanted at the time of the disaster.” Using qualitative descriptive analysis, we extracted the information on the support they wanted and then categorized and labeled them. Figure 1 shows the qualitative descriptive analysis procedure.

### 2.3. Ethical Considerations

The present study was approved by the institutional ethics committee of the National Institute of Health and Nutrition (Current National Institutes of Biomedical Innovation, Health and Nutrition), Japan (approval number: “20120626-05”). 

## 3. Results

Questionnaires were sent to 1911 RDs; 435 completed the questionnaire (response rate, 22.8%). Three hundred thirty-two participants (76.3%) responded to the open-ended question “Please write freely about the support that you wanted at the time of the disaster.” Table 1 shows that more females responded to the question (89.8%). The age breakdown of the respondents is as follows: 13.0% were 20–29 years, 27.1% were 30–39 years, 25.9% were 40–49 years, 24.4% were 50–59 years, 7.8% were 60–69 years, and 0.9% were 70–79 years. The highest proportion in the occupation category reported was welfare facilities (30.7%), while the second highest was hospitals (28.3%). 

**Figure 2:**
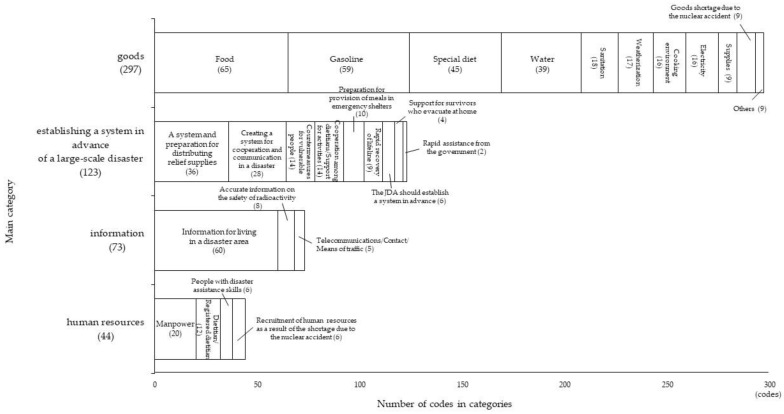
Number of main categories and categories. (): Number of codes.

The respondents’ necessary support was classified into four main categories: (1) “goods,” (2) “establishing a system in advance of a large-scale disaster,” (3) “information,” and (4) “human resources.” Figure 2 shows the main categories and categories. 

### 3.1. The Main Category: “Goods”

Table 2 shows categories delineated by the qualitative descriptive analysis within the main “goods” category, along with specific code examples and the number of codes supporting each category. Eleven categories were identified within the main category “goods”: “food” (65 codes), “gasoline” (59 codes), “special diet” (45 codes), “water” (39 codes), “sanitation” (18 codes), “weatherization” (17 codes), “cooking environment” (16 codes), “electricity” (16 codes), “supplies” (9 codes), “goods shortage due to the nuclear accident” (9 codes), and “others” (4 codes). 

The largest category was “food” (65 codes). Because the survey’s participants were RDs, many answers indicated that they desired nutrient-rich supplements difficult to acquire in emergency shelters. Code examples include “vegetables” (10 codes), “food to supplement nutrients” (5 codes), “protein source” (5 codes), etc. In addition, they requested food that could be provided in emergency shelters that did not have cooking facilities: “mass feeding for survivors” (4 codes), “cookless food” (4 codes), “canned food” (2 codes), etc. 

The second largest category was “gasoline” (59 codes). Survey answers mentioned a shortage of goods due to a shortage of gasoline. Specific code examples include “Even if there were relief supplies, I had no way to go there (gasoline)” and “In order to procure foods or relief goods, it was helpful if their arrangement to get gasoline was a priority.”

The third largest category was “special diet” (45 codes). The following responses specifically touched upon this: “It was difficult to deal with forms of food other than regular meals (soft, mixed, or minced) because the machine could not be used due to blackout” and “Enteral nutrition (concentrated liquid diet) was in short supply about 1 month after the earthquake, but there was no support for it.” Additionally, there were many responses relating to survivors with special dietary needs, for example, “My 1-year old daughter has an egg allergy, so I was at a loss for her diet. I was afraid to eat the meal to be distributed and was avoiding it” and “There were few supplies that could be provided as fortified food to people with kidney disease, heart disease, liver disease, and diabetes.”

In addition, there were 17 answers regarding weather-related support because the disaster occurred in a relatively cold area and season.

### 3.2. The Main Category: “Establishing a System in Advance of a Large-Scale Disaster”

Table 3 shows categories delineated by the qualitative descriptive analysis within the main “establishing a system in advance of a large-scale disaster” category, along with specific code examples and the number of codes supporting each category. The nine categories delineated within the main category “establishing a system in advance of a large-scale disaster” include “A system and preparation for distributing relief supplies” (36 codes), “Creating a system for cooperation and communication in a disaster” (28 codes), “Countermeasures for vulnerable people” (14 codes), “Cooperation among dietitians/Support for activities” (14 codes), “Preparation for provision of meals in emergency shelters” (10 codes), “Rapid recovery of lifeline” (9 codes), “The JDA should establish a system in advance” (6 codes), “Support for survivors who evacuate at home” (4 codes), and “Rapid assistance from the government.”.

The subcategory “A system for distributing relief supplies in a fair and appropriate quantity and timing” received the greatest number of responses (34 codes). The category “Countermeasures for vulnerable people” included “Appropriate response to vulnerable people” (8 codes) and “A system for delivering special diet to people who need it” (6 codes). “Nursing stations or nursing room (in emergency shelters)” and “A system support survivors unable to raise their voices,” which corresponds to vulnerable people, were also mentioned under the main category “goods.” The category “Cooperation among dietitians/Support for activities” featured the following answer: “It is necessary for dietitians to create a system that is positioned similarly to other occupations.” Due to the dispatch of dietitians to the disaster area for the first time, there were also many inquiries about creating a system in which dietitians could perform as dietitians.

### 3.3. The Main Category: “Information”

Table 4 shows categories delineated by the qualitative descriptive analysis within the main “information” category, along with specific code examples and the number of codes supporting each category. The three categories delineated within the main category “information” are: “Information for living in a disaster area” (60 codes), “Accurate information on the safety of radioactivity” (8 codes), and “Telecommunications/Contact/Means of traffic” (5 codes).

The category “Information for living in a disaster area” offered multiple answers suggesting that people wanted the necessary information to live in a disaster area and to receive support, for example, “I was worried that information on the damage situation would not come” and “Information on the neighborhood. Opening hours of gas stations and stores. Information on the water station (I got the upper limit per person after arriving).”

### 3.4. The Main Category: “Human Resources”

Table 5 shows categories delineated by the qualitative descriptive analysis within the main “human resources” category, along with specific code examples and the number of codes supporting each category. Four categories were delineated within the main category “human resources”: “Manpower” (20 codes), “Dietitian/Registered dietitian” (12 codes), “People with disaster assistance skills” (6 codes), and “Recruitment of human resources as a result of shortage due to the nuclear accident” (6 codes). 

The category “Manpower” received the most responses, with the next most frequently given being “Dietitian/Registered dietitian.” In addition, some participants responded that dietitians who were in a position to assist survivors needed a support person.

### 3.5. The Main Category: “Others”

Answers that could not be classified into the above four main categories were classified as “others.” Table 6 shows the specific code examples included in “others.”

## 4. Discussion

In order to document the necessary support for RDs in the areas affected by the earthquake, this study analyzed the written responses of dietitians to the open-ended question “Please write freely about the support that you wanted at the time of the disaster.” The responses were classified into four main categories: (1) “goods,” (2) “establishing a system in advance of a large-scale disaster,” (3) “information,” and (4) “human resources.”

### 4.1. The Main Category: “Goods”

Most of this category was essential to life, including “food,” “special diet,” and “water.” In the category “special diet,” many responses referred to “food for persons having difficulty in chewing/swallowing” and “concentrated liquid diet.” Oral health issues were often related to “difficulty swallowing” and “difficulty chewing” in the areas affected by the Great East Japan Earthquake [14]. One of the reasons behind these is that governments have few special food stockpiles reserved for such disasters. In the national survey of local governments, only 33.7% stated that special food stockpiles were included in their regional disaster prevention plans, guidelines, manuals, etc. [15]. The actual stockpiling rate of the local governments is even lower. Their rate was only 4.5% for the stockpiling of “food for persons having difficulty in chewing/swallowing.” This result reveals that only 19 local governments (1.5%) stockpile “food for persons having difficulty in chewing/swallowing” among all of the local governments that responded (*n* = 1272). It can therefore be concluded that vulnerable people should stockpile a sufficient quantity of special diet in order to protect themselves in the face of a disaster. In addition, it has been reported that prefectures that had previously been affected by disasters were likely to have food supply damage reporting systems [16]; therefore, establishing such a system might be a useful tool.

Many RDs responded concerning a need for “gasoline” (*n* = 59). Because the disaster areas were areas where cars were usually essential for transportation, it is thought that this shortage of “gasoline” impeded the distribution of supplies, contributing to the shortage of goods such as “food.” According to a previous report, “the large logistics companies or cooperatives often have in-tanks (small gas stations on their site), but their stockpiles are limited. They had an amount for only 2 or 3 days, and there were many cases of lack of supply. As for businesses that transport emergency relief supplies, although fuel was supplied preferentially, many businesses had difficulty securing it” [17]. In this analysis, there are also responses that “it was helpful if there were arrangements to make getting gasoline a priority,” and it was demonstrated that there was a shortage of fuel including gasoline. A previous review paper indicated that to deal with the challenges of large-scale disasters, we need to change many basic assumptions that we usually use in traditional business logistics [18]. It is necessary to make rebuilding the system that supplies the fuel a priority, especially in places where a lot of survivors depend on suppliers of goods, emergency shelters, and facilities for the elderly. 

### 4.2. The Main Category: “Establishing a System in Advance of a Large-Scale Disaster”

This main category included “A system and preparation for distributing relief supplies” and “Creating a system for cooperation and communication in a disaster.” In fact, we reported that “simulation and system maintenance both in the dispatch of RDs and in disaster areas is essential” [11]. It is possible to perform more appropriate support activities by making an assumption at the time of the disaster and preparing the system before the disaster occurs. In this analysis, one answer suggested that “training needs to be quick-witted.” In regard to this point, the JDA established “The Japan Dietetic Association-Disaster Assistance Team (JDA-DAT)” [7]. The JDA-DAT is a nutrition assistance team that received training in the specialty of nutrition care activities in disaster areas. The purpose of this team is to promote and foster RDs equipped with the necessary technical knowledge and techniques required in order to provide swift emergency dietary assistance for a disaster-stricken region in cooperation with medical, welfare, and government nutrition specialists, etc., when a large-scale natural disaster, such as a major earthquake or typhoon, occurs.

In this main category, one affected prefecture differed from the others. Many RDs who lived in Fukushima responded concerning a need for “creating a system for cooperation and communication in a disaster.” Compared to other prefectures, Fukushima was affected by a cascading disaster that the earthquake and tsunami triggered the meltdown of the Fukushima Daiichi nuclear reactor. The suffering of the survivors was not limited to the increased physical health problems directly attributable to radiation exposure but also included psychological and social effects [19]. The prevalence of mental disorders was high overall in Fukushima survivors [20]. Many residents were also subjected to home evacuation, displacement, relocation, work/school changes, and family separation [19,21]. In addition, they had raised concerns about the safety of food and water [22]. In areas affected by large-scale cascading disasters such as Fukushima, “creating a system for cooperation and communication in a disaster” is important for solving these problems. Effective dispatch of disaster dietitians (such as the JDA-DAT) is expected.

### 4.3. The Main Category: “Information”

Almost all answers in this main category mentioned “information for living in a disaster area” (*n* = 60). It is assumed that even “information about the neighborhood” was difficult to obtain, as the blackout limited communication and the lack of gasoline made transportation difficult. It has also been reported that “communication was difficult because means of sharing information could not be secured” and “accurate information could not be transmitted fairly and managed even as time passed from the occurrence of the disaster” [17]. Facilities that were reachable by landline, mobile phone, fax, or email had favorable dietary conditions [23]. Therefore, it is important to identify successful ways to obtain information at the time of disaster before disasters happen and to have communication devices that can be used in times of disaster.

### 4.4. The Main Category: “Human Resources”

In this main category, the need for human resources to assist with activities such as cooking, carrying water, etc., was the highest. In fact, the number of survivors dedicated to mass feeding assistance was higher as compared to the Self-Defense Forces and volunteers during the disaster [24]. It is essential to create a quick and effective system involving the assistance of an outside team, such as the Self-Defense Forces, volunteers, and dietitians.

The next most common answer was “dietitian/registered dietitian.” The usefulness of RDs at emergency shelters has been reported [10]. Additionally, there were some answers that requested “people with disaster assistance skills.” The JDA-DAT includes human resources who have both of these skills. Although the JDA-DAT has already been dispatched in response to some disasters in Japan, it is recommended that programs continue to prepare and foster disaster dietitians in preparation for future disasters. 

### 4.5. Limitations

One limitation of this study was that only 22.8% of the distributed questionnaires were returned. The disaster damage did not occur in the entire prefecture and affected only parts of it. Therefore, it is possible that dietitians who did not assist in the disaster area did not answer the questionnaire. However, the sample is considered to have possibly represented the Japanese RDs population because the results of previous studies targeting RDs all over Japan were similar in terms of sex ratio and occupation category to those in this study [25].

## 5. Conclusions

The findings suggest that a “system” is needed to ensure that “goods,” “information,” and “human resources” can easily and quickly enter a disaster area to facilitate nutritional support, specifically, (1) goods: creating a system to stock emergency supplies and deliver them where needed (a shift in assumptions that we usually use in traditional business logistics is also necessary [18]); (2) information: establishing a plan or system to obtain information at the time of disaster prior to a disaster occurring; and (3) human resources: establishing a system for dispatching volunteers and disaster dietitians (such as the JDA-DAT) in the appropriate places based on their specialties (the usefulness of RDs at emergency shelters has been reported [10]).

It is expected that each country will successfully implement the 2015 Sendai Framework for Disaster Risk Reduction (SFDRR). Our suggested systems are the result of a scientific analysis of the support actually needed in the disaster area. Therefore, establishing these systems is considered helpful for policy making to achieve disaster risk reduction in each country. In addition, it is essential to run a practical simulation prior to a disaster, making the best use of those “systems” in order to provide the support that is really needed in a large-scale disaster. Training on and practicing this system might also lead to disaster risk management. Indeed, the SFDRR have reported the following four renewed priorities: “It is important to promote regular disaster preparedness, response, and recovery exercises with a view to ensuring rapid and effective response to disasters and related displacement essential food and non-food relief supplies” [26]. Further, a systematic review reported that all four priorities for action are relevant to Disaster Risk Management [27]. 

It is necessary to establish a “system” to provide “goods,” “information,” and “human resources” to a disaster area in order to align practice and policy making based on the circumstances of each country in addition to scientific evidence.

## Figures and Tables

**Table 1:** Participants’ characteristics and responses (*n* = 332).

Characteristics		*n*	(%)
Sex			
	Male	6	(1.8)
	Female	298	(89.8)
	Unknown	28	(8.4)
Age			
	20–29 years	43	(13.0)
	30–39 years	90	(27.1)
	40–49 years	86	(25.9)
	50–59 years	81	(24.4)
	60–69 years	26	(7.8)
	70–79 years	3	(0.9)
	Unknown	3	(0.9)
Occupation category			
	Welfare facility	102	(30.7)
	Hospital	94	(28.3)
	Government	44	(13.3)
	School	25	(7.5)
	Education/Research	15	(4.5)
	Community activities	15	(4.5)
	Feeding facility	6	(1.8)
	Other dietitian positions	9	(2.7)
	Other occupations	6	(1.8)
	No work	14	(4.2)
	Unknown	2	(0.6)
Damage of workplace			
	None	99	(29.8)
	Partially damaged	181	(54.5)
	Completely destroyed	9	(2.7)
	Unknown	43	(13.0)

**Table 2:** Category, subcategory, and specific code examples included in the main category “goods” (Codes total: *n* = 297).

Category (Number of Codes)	Subcategory (Number of Codes)	Number of Codes and Specific Code Examples (Per Each Prefecture)		
		Iwate Prefecture	Miyagi Prefecture	Fukushima Prefecture
Food (65)	Food (25)	6 codes -Dietary support for living rather than nutritional counseling was important in the situation without food. -Preparing stockpile food for staff.	12 codes -Food did not reach us until day 7, and I was at a loss. -Ingredients of rice ball	7 codes -Food shortages occurred in disaster areas and areas where survivors were accepted. -There was a limit to the number of ingredients I could buy.
	Vegetables (10)	1 code -Vegetables	4 codes -Vegetables	5 codes -Bread was provided as a relief supply, but survivors were not very pleased. Vegetables were few, and volunteers brought them.
	Food to supplement nutrients (5) (e.g., dietary supplements)	3 codes -The dietary supplement arrival was late. -Something to supplement nutrition-deficient nutrients such as vitamins.	1 code -Even if relief supplies arrived, it was only bread or noodles. Micronutrients were not supplied.	1 code -There were a lot of rice balls and bread at emergency shelters. Many survivors seemed to be constipated.
	Protein source (5)	–	1 code -Protein source	4 codes -Perishable foods (protein sources such as fish and meat) -Retort food to be protein sources -Milk
	Rice/Bread (4)	2 codes -Rice/Bread	–	2 codes -Rice -Bread
	Mass feeding for survivors (4)	–	2 codes -A high frequency of mass feeding for survivors was better than supplying the supplement. -Support for “Hot meal mass feeding for survivors”	2 codes -Mass feeding for survivors (this differs among respective emergency shelters)
	Cookless food (4)	2 codes -PET bottle of Japanese tea	1 code -Ready-to-eat food	1 code -Cookless food
	Canned food (2)	2 codes -Canned food	–	–
	Seasoning (2)	1 code -Salt, soy sauce, miso, and sugar	–	1 code -Spices such as ginger, garlic, or green onion
	Luxury food (2)	1 code -Luxury food such as coffee	–	1 code -Something to drink (milk, Yakult, or others)
	Retort pouch food (1)	1 code -Retort pouch food	–	–
	Food with long storage period (1)	–	1 code -Food with long storage period	–
Gasoline (59)	Gasoline (40)	14 codes -It was inconvenient because the gasoline shortage continued and I could not use my car.	9 codes -Gasoline	17 codes -There was a shortage of gasoline in the disaster areas and the areas that accepted survivors. -Gasoline was the most necessary because it was an area where I could not move around without a car.
	Gasoline for commuting (9)	2 codes -There were times when I could not go to work because gasoline was running short.	1 code -Gasoline for commuting	6 codes -Because of a gasoline shortage, it was difficult to secure our staff. I desired an oil tank.
	Gasoline for manufacturers to transport goods (6)	5 codes -I did not receive deliveries due to a gasoline shortage. -The stoppage of distribution due to a gasoline shortage caused confusion of information.	–	1 code -Because of a gasoline shortage, there were approximately three companies that were unable to deliver.
	Gasoline to go shopping/to go pick up goods(4)	–	2 codes -Even if there were relief supplies, I had no way to go there (gasoline). -In order to go pick up foods or relief supplies, it would be helpful if arrangement to get gasoline was a priority.	2 codes -Gasoline to go shopping
Special diet(45)	Food for a person having difficulty in chewing/swallowing (15)	7 codes -It was difficult to deal with forms of food other than regular meals (soft, mixed, or minced) because the machine could not be used due to blackout. -Rice porridge	4 codes -High-calorie meals in a facility for the elderly	4 codes -Food for persons with difficulty swallowing (because there was a shortage, we provided less quantity). -We alternated shifting from concentrated liquid diets to medicine.
	Concentrated liquid diet (14)	7 codes -Enteral nutrition (concentrated liquid diet) was in short supply about 1 month after the earthquake, but there was no support for it. -Distribution of concentrated liquid diet was reduced, so it was not certain when and how many would be delivered.	4 codes -Jelly for hydration in a facility for the elderly	3 codes -Concentrated liquid diet
	Fortified food (7)	2 codes -Fortified food	2 codes -Fortified food	3 codes -Fortified food
	Food for infants (4)	4 codes - Milk powder -Infant food in the early phase after the disaster -Water for infant in the early phase after the disaster	–	–
	Hypoallergenic food (3)	–	1 code -My 1-year-old daughter has an egg allergy, so I was at a loss for her diet. I was afraid to eat the meal to be distributed and was avoiding it.	2 codes -Initially, we could not be deal with survivors with meal problems (e.g., food allergy).
	Food for patients (2)	–	1 code -Low-salt diet	1 code -There were few supplies that could be provided as fortified food to people with kidney disease, heart disease, liver disease, and diabetes.
Water (39)	Water/Water truck (30)	8 codes -Procurement of water was difficult. A water truck came on day 4. Still, staff went to draw water from the purification plant.	8 codes -Permanent water truck -I got in the line for the water truck because I had little water stocked. But I had a hard time carrying 2 L of water per person.	14 codes -We had to secure water ourselves and did not receive water supply assistance. -To procure water, facility staff had to leave to procure water several times a day.
	Drinking water/Water for the meal (4)	–	2 codes -Drinking water -Water for the meal	2 codes -Drinking water or oral rehydration solution such as OS-1 (Dehydration did not occur because it was in winter, but it would have been serious if it was in summer.)
	Cleaning water (2)	–	2 codes -Cleaning water except for drinking water -It was difficult to secure water, so it was a challenge to wash dishes and cooking tools.	–
	Water for toilet (2)	–	–	2 codes -A limit was set to water in the toilet. Considering sanitation, we needed water support.
	Water for bath (1)	–	–	1 code -I could not take a bath because water was not coming out at my house.
Sanitation (18)	Disposable tableware and cling film (14)	2 codes -Disposable tableware, cling film and polyethylene bags	10 codes -I prepared disposable tableware, but it was an issue because disposable dishes were running out quickly due to water failure.	2 codes -Disposable tableware/cling wrap
	Hygiene (2)	1 code -Hygiene	1 code -Hygiene	–
	Portable toilet/Diapers (2)	1 code - Diapers	1 code -Portable toilet	–
Weatherization (17)	Kerosene (7)	6 codes -Kerosene (Because it was cold.)	–	1 code -Kerosene
	Blanket/Bedding/Clothes (4)	1 code -Blanket	3 codes -Bedding (Especially a mattress. I slept directly on the floor, and I thought that my back would hurt and it would cause bedsores.) -Clothes	–
	Heat source (3)	–	3 codes -Heat source	–
	Heater (2)	1 code -Heater (1)	–	1 code -Heater and fuel for emergency
	Stove (1)	–	1 code -Kerosene stove	–
Cooking environment (16)	Gas (7)	1 code -Gas cylinder for heating	5 codes -Measures to secure heating source (especially necessary for cooking)	1 code -Support of gas
	Gas stove (5)	2 codes -Gas stove	1 code -I wanted a gas stove that can use propane gas because the heat source was insufficient.	2 codes -I wanted a cooking stove because I was not able to use gas.
	Cooking equipment/Cooking tool (4)	–	1 code -Cooking tool	3 codes -Cooking equipment -Refrigerator
Electricity (16)	Electricity/Electric power (11)	4 codes -Because home appliances are all-electric, I could not cook, wash, and disinfect during a blackout. So, I was concerned about food poisoning.	6 codes -Even if information is obtained on the Internet, printing is impossible (electricity, paper, and machine). -Electric power for using cooking tools	1 code -Electricity/Electric power
	Battery (3)	1 code -Generator (including battery worked by hand)	2 codes -Battery	–
	Lighting (1)	–	1 code -Lighting	–
	Candle (1)	1 code -Lighting (candle)	–	–
Supplies (9)	Supplies from the government (5)	–	–	5 codes -Supplies from the government
	Supplies (4)	–	2 codes -Supplies	2 codes -I desired much support from Western Japan.
Goods shortage due to the nuclear accident (9)	Food shortage due to the nuclear accident (3)	–	–	3 codes -Because of a rumor, it was awkward that I could not have food delivered (concentrated liquid diet) in Fukushima.
	Supply shortage due to the nuclear accident (3)	–	1 code -Because our facility was 20~30 km from the nuclear power plant, supplies were not distributed.	2 codes -Supply shortage due to the nuclear accident
	Safe water due to radioactive contamination (2)	–	–	2 codes -As water was contaminated with radioactivity, water became increasingly precious. Water was needed for dialysis as well as for cooking.
	Gasoline shortage due to the nuclear accident (1)	–	–	1 code -Gasoline shortage due to the nuclear accident
Others (4)	Daily commodities/Concerning housing (2)	1 code -Daily commodities/Concerning housing	1 code -Earthquake resisting device is necessary.	–
	Communication equipment that can be used even in emergency (1)	–	–	1 code -Communication equipment that can be used even in emergency
	Money (1)	–	–	1 code -Money to buy what I need for cooking other than relief supplies.

**Table 3:** Category, subcategory, and specific code examples included in the main category “establishing a system in advance of a large-scale disaster” (Codes total: *n* = 123).

Category (Number of Codes)	Subcategory (Number of Codes)	Number of Codes and Specific Code Examples (Per Each Prefecture)		
		Iwate Prefecture	Miyagi Prefecture	Fukushima Prefecture
A system and preparation for distributing relief supplies (36)	A system for distributing relief supplies in a fair and appropriate quantity and timing (34)	4 codes -A system for distributing relief supplies in a fair and appropriate quantity and timing	14 codes -Even if we were able to harvest vegetables, etc., we could not open the market and could not adjust our distribution.	16 codes -My desired goods could not be obtained as I expected (there were few vegetables, and there were many processed foods that were not versatile foods).
	Stockpile (2)	1 code -Stockpile	1 code -Stockpile	–
Creating a system for cooperation and communication in a disaster (28)	Creating a system for cooperation and communication in a disaster (28)	5 codes -Creating a system for cooperation and communication in a disaster	8 codes -It is necessary to cooperate daily and to train so that information can be utilized at all times.	15 codes -Creating a system for cooperation and communication in a disaster
Countermeasures for vulnerable people (14)	Appropriate response to vulnerable people (8)	2 codes -Even though it required energy, everyone was fair and personal responses were few.	4 codes -Nursing stations or nursing room (in emergency shelters) -Correspondence of people who are eating a lot of processed foods.	2 codes -A system to support survivors unable to raise their voices.
	A system for delivering special diet to people who need it (6)	–	4 codes -People with food allergies (especially wheat) returned to their homes early because they could not eat from stockpiles.	2 codes -Only food for general use was distributed. I wanted them to consider that there are infants, children, mildly sick people, and elderly people in emergency shelters.
Cooperation among dietitians/Support for activities (14)	A system for cooperation among dietitians (8)	3 codes -I wanted continued support for 1 week at least.	3 codes -A system for cooperation among dietitians	2 codes -Information tool for communication among neighbor dietitians
	Activity contents/place as a dietitian (6)	2 codes -It is necessary for dietitians to create a system that is positioned similarly to other occupations.	3 codes -Support to serve as a dietitian. We cannot act arbitrarily in the government (dietitian’s work is not described in the disaster prevention plan, manual, etc.).	1 code -I was disappointed that there was no contact from the JDA even after 1 month (other professional associations provided support information including volunteers every day).
Preparation for provision of meals in emergency shelters (10)	Cooking environment in emergency shelter without lifeline (5)	1 code -Cooking environment in emergency shelter without lifeline	3 codes -It was necessary for dietitians to make rounds in each emergency shelter to advise about sanitation and taking turns cooking.	1 code -Cooking environment in emergency shelter without lifeline
	Menu suggestions for meals in emergency shelters without a gas and/or water supply (5)	1 code -Menu suggestions for meals in emergency shelters without a gas and/or water supply	3 codes -Menu suggestions for meals in emergency shelters without a gas and/or water supply	1 code -Menu suggestions for meals in emergency shelters without a gas and/or water supply
Rapid recovery of lifeline (9)	Rapid recovery of lifeline (9)	–	6 codes -Lifeline recovery was slow, and it was hard to cook.	3 codes -I think that there was not enough water, and its recovery was a little late.
The JDA should establish a system in advance (6)	The JDA should establish a system in advance (6)	1 code -Even as the JDA, we need a position to assist cooking, etc., immediately.	3 codes -Skill to prepare for independent management day to day. Training to be quick-witted. -It would be helpful if the JDA could coordinate food provision. -Booklet that can be kept at hand.	2 codes -The JDA should build a network that can promptly share information (e.g., add e-mail address at the time of member registration). -Even as part of the JDA, it was necessary to go around directly to the hospitals or to listen to their comments from the site.
Support for survivors who evacuate at home(4)	Create a support system for survivors who evacuate at home (4)	1 code -It was difficult to extend relief supplies to survivors who evacuated to their own homes.	1 code –Correspondence to survivors who evacuated at home	2 codes -There was no nutrition assistance for survivors with sick who evacuated at their homes. -It was better to have support in the form of public relations about how to eat because people had mostly carbohydrate-rich meals at home.
Rapid assistance from the government (2)	Rapid assistance from the government (2)	1 code -Rapid assistance from the local government	–	1 code -Rapid assistance from the government

**Table 4:** Category, subcategory, and specific code examples included in the main category “information” (Codes total: *n* = 73).

Category (Number of Codes)	Subcategory (Number of Codes)	Number of Codes and Specific Code Examples (Per Each Prefecture)		
		Iwate Prefecture	Miyagi Prefecture	Fukushima Prefecture
Information for living in a disaster area (60)	Information (30)	8 codes -Information (I spent about 3 days without knowing anything because the newspaper did not come due to the blackout.)	18 codes -I was worried that information on the damage situation would not come. -Almost no information obtained. I should have checked where and what information I could obtain.	4 codes -Support to make up for lack of information
	Details of support contents received (8)	2 codes -Details of support contents received	2 codes -Details of support contents received	4 codes -It was very difficult to obtain foodstuffs from our outsourcing company. I wanted information as to where I could find special diet that was available.
	Information on the neighborhood (8)	1 code -Information on the neighborhood	2 codes -I wanted to join the support if I could get information. -I could not understand the situation of disaster. The reason is that I was unable to move around because the surrounding area was covered with water due to the tsunami. I wanted information.	5 codes -Information on the neighborhood. Opening hours of gas stations and stores. Information on the water station (I got the upper limit per person after arriving). -Information on other areas because I was unable to think about the situation of other areas.
	Accurate information (7)	2 codes -Accurate information	–	5 codes -Accurate information
	Information from the government (5)	–	1 code -Information from the government	4 codes -I wanted a clear response that the government is unable to assist us. I could get no response from the government, even though they asked us about our troubles and needs because the facility side just said that and the government side just listened.
	Recovery information on lifeline (2)	–	–	2 codes -Recovery information on lifeline
Accurate information on the safety of radioactivity (8)	Information on the effects of radioactivity on food and drinks and the action to be taken (6)	–	–	6 codes -Accurate information on the safety of radioactivity (some people asked for advice about the following: They inquired with the national counseling desk but the answers varied according to each organization). -After becoming aware of the nuclear accident, I was very anxious that it was not good to eat the stored root vegetables. I was dependent on only instant foods for a while because the store was closed.
	Accurate information on the nuclear accident (2)	–	–	2 codes -Accurate information on the nuclear accident
Telecommunications/Contact/Means of traffic (5)	Telecommunications/Contact/Means of traffic (5)	2 codes -Telecommunications/Contact/Means of traffic	3 codes -Communication system with other facilities	–

**Table 5:** Category, subcategory, and specific code examples included in the main category “human resources” (Codes total: *n* = 44).

Category (Number of Codes)	Subcategory (Number of Codes)	Number of Codes and Specific Code Examples (Per Each Prefecture)		
		Iwate Prefecture	Miyagi Prefecture	Fukushima Prefecture
Manpower (20)	Manpower (14)	1 code -I wanted a support person for paperwork.	7 codes -In the case of the government, it is extremely difficult to maintain management of emergency shelters concurrently. I wanted physical support for emergency shelters from an early phase.	6 codes -Manpower carrying water with polyethylene tanks -Elderly people’s hands were very full. Many people injured their lower backs due to carrying water.
	Human resources for mass feeding/cooking (3)	3 codes -Human resources for mass feeding/cooking	–	–
	Human resources for mental support (2)	2 codes -Human resources for mental support	–	–
	Human resources to distribute the relief supplies (1)	–	1 code -Human resources to distribute the relief supplies	–
Dietitian/Registered dietitian (12)	Rapid dispatch of dietitian/registered dietitian (12)	3 codes -Dietitian staffing is needed for the relief supply center.	4 codes -It was late to start officially dispatching dietitians.	5 codes -Member who can participate at all times (weekdays)
People with disaster assistance skills (6)	People with disaster assistance skills (6)	1 code -A place to respond to when we are in trouble.	4 codes -People who brainstorm together regarding how to assist the survivors	1 code -I think that it will be necessary to have competent support with coordinating support activities while identifying priority issues with the local government.
Recruitment of human resources as a result of the shortage due to the nuclear accident (6)	Recruitment of human resources as a result of the shortage due to the nuclear accident (6)	–	1 code -I was not able to express that I wanted support to come because it was close to the Fukushima Daiichi nuclear disaster area.	5 codes -Because of Fukushima prefecture, the support was mainly goods. It was not a troubling thing, but I wanted the support of personnel more. The staff was also evacuated, so there was a human resource shortage.

**Table 6:** Category, subcategory, and specific code examples included in the main category “others” (Codes total: *n* = 30).

Category (Number of Codes)	Subcategory (Number of Codes)	Number of Codes and Specific Code Examples (Per Each Prefecture)		
		Iwate Prefecture	Miyagi Prefecture	Fukushima Prefecture
Nothing special (25)	Nothing special (25)	10 codes -It never crossed my mind to demand someone in particular. -It did not cause a major inconvenience for residents of the facility (in Morioka City). -Mass feeding offered to survivors by the local government were helpful.	7 codes -I cannot recall because time has passed. -My family managed to get everything done somehow by ourselves.	8 codes -Because I was provided with enough support, it was a great help to me. -The foods were all right, thanks to the efforts of suppliers. -Support was not particularly necessary because no great damage occurred.
Others (5)	Others (5)	–	3 codes -I was not able to be active because I was busy with housework and work and missing my family. -It helped us to prepare various things, thanks to the seminar by the Miyagi dietetic association before the earthquake. -This earthquake made me deeply understand that diet can save a life.	2 codes -It was a priority to provide our meals because our hospital was completely destroyed.
